# Hybridization patterns between two marine snails, *Littorina fabalis* and *L. obtusata*

**DOI:** 10.1002/ece3.5943

**Published:** 2020-01-21

**Authors:** Diana Costa, Graciela Sotelo, Antigoni Kaliontzopoulou, João Carvalho, Roger Butlin, Johan Hollander, Rui Faria

**Affiliations:** ^1^ CIBIO‐InBIO Centro de Investigação em Biodiversidade e Recursos Genéticos Universidade do Porto Vairão Portugal; ^2^ Department of Biology Faculty of Sciences University of Porto Porto Portugal; ^3^ CIIMAR Interdisciplinary Centre of Marine and Environmental Research University of Porto Porto Portugal; ^4^ cE3c Centre for Ecology, Evolution and Environmental Changes Departamento de Biologia Animal Faculdade de Ciências da Universidade de Lisboa Lisbon Portugal; ^5^ Department of Animal and Plant Sciences University of Sheffield Sheffield UK; ^6^ Department of Marine Sciences University of Gothenburg Gothenburg Sweden; ^7^ Department of Biology Aquatic Ecology Unit Lund University Lund Sweden; ^8^ Global Ocean Institute World Maritime University Malmö Sweden

**Keywords:** ecological speciation, ecotypes, flat periwinkles, gene flow, geographic context, introgression, male genitalia, natural selection, reproductive isolation, shell morphology

## Abstract

Characterizing the patterns of hybridization between closely related species is crucial to understand the role of gene flow in speciation. In particular, systems comprising multiple contacts between sister species offer an outstanding opportunity to investigate how reproductive isolation varies with environmental conditions, demography and geographic contexts of divergence. The flat periwinkles, *Littorina obtusata* and *L. fabalis* (Gastropoda), are two intertidal sister species with marked ecological differences compatible with late stages of speciation. Although hybridization between the two was previously suggested, its extent across the Atlantic shores of Europe remained largely unknown. Here, we combined genetic (microsatellites and mtDNA) and morphological data (shell and male genital morphology) from multiple populations of flat periwinkles in north‐western Iberia to assess the extent of current and past hybridization between *L. obtusata* and *L. fabalis* under two contrasting geographic settings of divergence (sympatry and allopatry). Hybridization signatures based on both mtDNA and microsatellites were stronger in sympatric sites, although evidence for recent extensive admixture was found in a single location. Misidentification of individuals into species based on shell morphology was higher in sympatric than in allopatric sites. However, despite hybridization, species distinctiveness based on this phenotypic trait together with male genital morphology remained relatively high. The observed variation in the extent of hybridization among locations provides a rare opportunity for future studies on the consequences of different levels of gene flow for reinforcement, thus informing about the mechanisms underlying the completion of speciation.

## Graphical abstract

The flat periwinkles, *Littorina obtusata* and *L. fabalis* (Gastropoda), comprise an interesting system to study how reproductive barriers evolve during (ecological) speciation with gene flow. In this study, we characterized the patterns of hybridization between the two intertidal sister species across multiple populations in the Iberian Peninsula, combining genetic and morphological data. By contrasting geographic settings of divergence, an extensive variation of hybridization among locations was found, opening doors to further studies on reinforcement and its role in speciation.

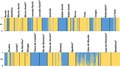

## 1 INTRODUCTION

It is widely accepted that the number of traits contributing to reproductive isolation generally increases as speciation progresses (Seehausen et al., 2014; Smadja & Butlin, 2011). However, how traits under different evolutionary forces such as sexual selection (Ritchie, 2007), natural divergent selection (Nosil, 2012), and selection against maladaptive hybridization (Butlin, 1987; Hollander et al., 2018) interact with each other toward completing speciation is still largely unknown.

Distinguishing whether sister species are or are not completely reproductively isolated is a key step to identify traits involved in speciation. Traits differentiating species that are fully reproductively isolated could have evolved after speciation was complete and have not necessarily contributed to reduce gene flow between them (Butlin, 1987; Coyne & Orr, 2004; Nosil & Schluter, 2011). For example, a recent study on mangrove snails, *Littoraria cingulata* and *L. filosa*, found reproductive character displacement in assortative mating, but no gene flow between the two species, suggesting that displacement was caused by reproductive interference after speciation was complete (Hollander et al., 2018). By contrast, sister species that hybridize can be an important source of knowledge about the buildup of traits that act as barriers during the progress of speciation (Abbott et al., 2013).

Although divergence in the presence of gene flow is a widely accepted mechanism (Pinho & Hey, 2010), our view of speciation remains oversimplified. Even when models of divergence take into account multiple rates of gene flow across the genome and through time (Roux et al., 2014; Sousa, Carneiro, Ferrand, & Hey, 2013), inferences about the genomic architecture of speciation often assume that there is one landscape of divergence across the genome that is consistent in all contacts between a pair of species. However, divergence between two evolutionary units often involves multiple geographical replicates (e.g., sticklebacks, Jones et al., 2012; rough periwinkles, Butlin et al., 2014) or a wide distribution with multiple opportunities for hybridization in contact zones that are relatively independent from each other (e.g., house mouse, Smadja, Catalan, & Ganem, 2004; fire‐bellied toads, Szymura & Barton, 1986). Thus, species interactions are likely to be context‐dependent both in space and time, resulting in different rates of hybridization with locality‐specific evolutionary consequences (Harrison & Larson, 2016). Such heterogeneity is particularly relevant to understand how different barrier traits accumulate and work in concert to strengthen reproductive isolation. The mechanisms completing speciation, such as reinforcement, depend not only on the opportunity for hybridization but also on its costs and benefits, which may vary idiosyncratically among contact zones. Studies of multiple replicates where sister species contact and have the opportunity to hybridize, but also comprising different environmental conditions, demographic scenarios, and geographic contexts of divergence, are thus needed to gain a more comprehensive understanding of how hybridization and barrier traits vary across the species’ range.

Marine gastropods of the family Littorinidae have been extensively studied in the context of ecological speciation (Johannesson, 2003; Rolán‐Alvarez, Austin, & Boulding, 2015). This includes the rough periwinkle *Littorina saxatilis*, for which several ecotypes have been evolving in parallel (Butlin et al., 2014; Johannesson, 2003; Reid, 1996), the *Littoraria* species mentioned above (Hollander et al., 2018), but also the sister species *Littorina fabalis* and *L. obtusata* (Carvalho, Sotelo, Galindo, & Faria, 2016; Kemppainen, Lindskog, Butlin, & Johannesson, 2011; Kemppainen, Panova, Hollander, & Johannesson, 2009; Rolán‐Alvarez et al., 2015; Sotelo et al., 2020). Commonly known as flat periwinkles, the latter two species started to diverge around 0.8–1.3 million years ago (Kemppainen et al., 2009; Marques et al., 2017 **;** Reid, Rumbak, & Thomas, 1996; Sotelo et al., 2020; Tatarenkov, 1995), showing high genetic differentiation in allozymes (Nei‘s interspecific vs. intraspecific genetic distance of 0.458 vs. 0.014, respectively; Rolán‐Alvarez, Zapata, & Alvarez, 1995) and microsatellite loci (*F*_ST_ > 0.3 for the majority of tested loci, Carvalho et al., 2015; mean *F*_ST_ = 0.45, Carvalho et al., 2016). However, widespread mtDNA haplotype sharing raised the hypothesis of gene flow during divergence between the two species (Kemppainen et al., 2009), which was supported by recent analyses (Sotelo et al., 2020). Together with the identification of several early generation hybrids in a single site close to the southern distribution limit of these species (Carvalho et al., 2016), the divergence between flat periwinkle species is compatible with an advanced stage of speciation, with some residual gene flow in the present and more extensive gene flow in the past.

These two sibling species present a largely overlapping distribution along the European Atlantic shores (Reid, 1996). However, at a local scale, pockets of allopatric populations can be found, especially in north‐western Iberia (Sotelo et al., 2020). Moreover, *L. obtusata* is usually found in more sheltered habitats than *L. fabalis*. These include areas of Galician bays (“Rías”) in Spain, as well as sites in northern Portugal that are somewhat protected from waves. *Littorina fabalis*, on the other hand, occupies more exposed areas with stronger wave action. In Iberian locations where the two species co‐occur (hereafter referred to as sympatric for simplicity), they tend to show some level of vertical zonation, with *L. obtusata* occupying the mid to upper part of the shore, while *L. fabalis* tends to inhabit the lower part.

Three *L. fabalis* ecotypes were previously identified in this region, facing different wave exposure regimes and dwelling in different macroalgae/seagrass. The Mastocarpus Exposed (ME) ecotype is usually found in exposed sites on *Mastocarpus* spp.; the Zostera Sheltered (ZS) ecotype is found in a single sheltered region associated with *Zostera* spp.; and the *Fucus* Intermediate (FI) ecotype is commonly found in *Fucus *spp. (Carvalho et al., 2016; Rolán & Templado, 1987). Some variation in shell morphology associated with wave exposure has also been described within *L. obtusata* (Reid, 1996). However, in contrast to *L. fabalis*, there is no association between this variation and macroalgae species. Since the phenotypic differences and distribution of these variants in Iberia have not been characterized in a systematic manner, the ecotype terminology is generally not used for *L. obtusata*.

Contact between *L. obtusata* and all different *L. fabalis* ecotypes has been observed during fieldwork for this and previous studies (Carvalho et al., 2016) but, given the distribution of both species, those involving the FI ecotype are the most common. How this diversity in terms of geographic context of divergence and local environmental conditions influences the prevalence of hybridization between *L. obtusata* and *L. fabalis* remains unclear.

Flat periwinkles also exhibit high intraspecific shell polymorphism in color patterns, as well as in size and shape (Rolán‐Alvarez et al., 2015). Although *L. fabalis* tends to be smaller and have a different shell shape (typically a rounder shell with a wider aperture) when compared to *L. obtusata* (typically a more elongated shell and smaller aperture relative to size), male genital morphology is the most reliable trait for distinguishing sister species (Reid, 1996). A comparative analysis across Littorininae revealed greater male genital shape divergence between sympatric/parapatric sister species when compared with allopatric pairs (Hollander, Smadja, Butlin, & Reid, 2013), with flat periwinkles standing out as a strong candidate for prezygotic isolation to have evolved as a consequence of hybridization. However, similar patterns could have resulted from reproductive interference to reduce direct costs associated with interspecific mating after reproductive isolation was complete (Hollander et al., 2018). Thus, the comparisons between populations with different levels of hybridization is a prerequisite for further tests of reinforcement or other processes leading to completion of reproductive isolation in this system.

Here we have analyzed multiple north‐western Iberian populations representing different geographical contexts (allopatric and sympatric) between flat periwinkles. We analyzed genetic data (microsatellites and mtDNA) together with shell and male genital morphology of snails from 27 Iberian sites in order to (a) characterize the extent of hybridization between the two sister species; (b) evaluate differences in hybridization frequency and dynamics across distinct geographic settings; and (c) assess the influence of hybridization on the phenotypic differences (shell and male genitalia morphology) between species across sites.

## 2 MATERIALS AND METHODS

### 2.1 Sampling of flat periwinkles

**Figure 1:**
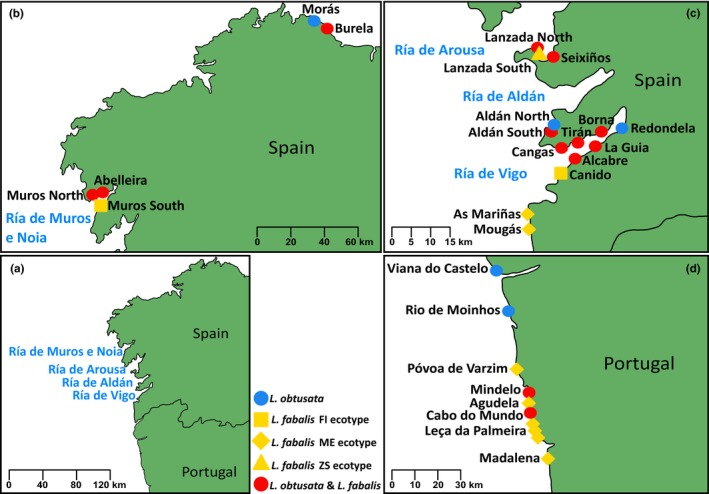
Sampling locations across the distribution range of *Littorina fabalis* and *Littorina obtusata* in the Iberian Peninsula. (a) General overview of the Iberian shore extent where the 27 sampled sites are distributed. (b) Zoom in of northern sampling sites. (c) Zoom in of sampling sites in Ría de Arousa and Ría de Aldán. (d) Zoom in of sampling sites in the Portuguese coast. Names of the sites follow those presented in Table 1

Sampling covered sites in the north‐western part of the Iberian Peninsula where both *L. fabalis* and *L. obtusata* were present (sympatric), as well as sites where only one species was found (locally allopatric) (Figure 1, Table 1). For the analyses requiring reference allopatric populations, these were chosen based on several field surveys performed in these locations over the years, where we only found one species. Since fully grown individuals were needed for morphological analyses, sampling efforts were directed toward adults. Otherwise, individuals were randomly collected in terms of shell shape and size to avoid biasing our sampling toward either of the species or potential hybrids. The ecotype of *L. fabalis* individuals was recorded based on the algae/seagrass where they were found. Individuals were collected at the lowest tides (<0.75 m) and were brought alive to the laboratory where they were frozen at −20°C.

**Table 1:** Information of the individuals analyzed in this study, displaying the locations sampled; date of collection; geographical context of the species distribution in each location; species present in each location (males classified into species based on shell appearance and penis morphology and females classified based on shell appearance); *Littorina fabalis* ecotype (when applicable); number of individuals collected (*N*); number of individuals analyzed for microsatelites (*N*_micros_); number of individuals analyzed for mtDNA (*N*_mt_); and number of individuals analyzed for shell morphology (*N*_GM_)

Location	Code	Collection date	Distribution	Species	Ecotype	*N*	*N*_micros_	*N*_mt_	*N*_GM_	Coordinates	
										Latitude	Longitude
1. Burela	BUR	October 2015	Sympatric	*Littorina fabalis* and *Littorina obtusata*	ME/FI^a^	34	15	15	20	43°39′60″N	7°21′23″W
2. Morás^f^	MOR	October 2015	Undetermined	*L. obtusata*	—	15	9	9	0	43°43′08″N	7°28′25″W
3. Abelleira	ABE	December 2012	Sympatric	*L. fabalis* and *L. obtusata*	FI	84	24^b^	18	31	42°47′53″N	9°01′30″W
4. Muros North	MURN	December 2012	Sympatric	*L. fabalis* and *L. obtusata*	FI	40	40	37	14	42°46′25″N	9°03′06″W
5. Muros South	MURS	December 2012	Allopatric	*L. fabalis*	FI	25	24^b^	13	19	42°44′34″N	8°58′53″W
6. Lanzada North	LANN	July 2015	Sympatric	*L. fabalis* and *L. obtusata*	ZS	95	44	39	30	42°27′52″N	8°52′02″W
7. Lanzada South	LANS	December 2012; February–March 2015	Allopatric	*L. fabalis*	ZS	78	23 b	22^d^	34	42°27′38″N	8°52′19″W
8. Seixiños	SEI	December 2012; March 2015	Sympatric	*L. fabalis* and *L. obtusata*	ZS	79	77^c^	51	38	42°27′28″N	8°49′23″W
9. Aldán North	ALDN	February 2015	Allopatric	*L. obtusata*	—	46	40	20	23	42°16′47″N	8°49′13″W
10. Aldán South	ALDS	February 2015	Sympatric	*L. fabalis* and *L. obtusata*	FI	20	20	18	8	42°16′36″N	8°49′29″W
11. Borna	BOR	July 2015	Sympatric	*L. fabalis* and *L. obtusata*	FI	66	42	42	24	42°16′51″N	8°41′49″W
12. Tirán	TIR	November 2012; July 2015	Sympatric	*L. fabalis* and *L. obtusata*	FI	147	49^c^	40^e^	36	42°15′49″N	8°45′16″W
13. Cangas	CAG	November 2012	Sympatric	*L. fabalis* and *L. obtusata*	FI	150	55^c^	42	49	42°15′21″N	8°47′16″W
14. Redondela	RED	July 2015	Allopatric	*L. obtusata*	—	57	40	23	36	42°17′15″N	8°37′22″W
15. La Guia	GUI	February 2013	Sympatric	*L. fabalis* and *L. obtusata*	FI	62	47	37	32	42°15′31″N	8°42′13″W
16. Alcabre	ALC	July 2015	Sympatric	*L. fabalis* and *L. obtusata*	FI	53	42	35	29	42°13′25″N	8°45′57″W
17. Canido	CAN	October 2012	Allopatric	*L. fabalis*	FI	93	24^b^	21	34	42°11′32″N	8°48′19″W
18. As Mariñas^g^	MAR	October 2012; February 2013	Allopatric	*L. fabalis*	ME	74	24^b^	19	0	42° 6′01″N	8°53′43″W
19. Mougás	MOU	November 2012	Allopatric	*L. fabalis*	ME	24	24^b^	20	0	42° 3′39″N	8°53′27″W
20. Viana do Castelo	VIA	September 2014	Allopatric	*L. obtusata*	—	60	39	20	36	41°41′45″N	8°51′02″W
21. Rio de Moinhos	MOI	November 2012	Allopatric	*L. obtusata*	—	85	35^b^	30^d^	36	41°34′00″N	8°47′50″W
22. Póvoa de Varzim	POV	November 2012	Allopatric	*L. fabalis*	ME	63	23^b^	21^d^	28	41°23′05″N	8°46′29″W
23. Mindelo	MIN	October 2015	Sympatric	*L. fabalis* and *L. obtusata*	ME	90	70	68	47	41°18′36″N	8°44′33″W
24. Agudela	AGU	November 2012	Allopatric	*L. fabalis*	ME	65	32	19	26	41°14′34″N	8°43′44″W
25. Cabo do Mundo	CMU	November 2012; September 2014; March 2015	Sympatric	*L. fabalis* and *L. obtusata*	ME/FI^a^	125	111^c^	101^e^	39	41°13′33″N	8°43′03″W
26. Leça da Palmeira^g^	LEC	March 2015	Allopatric	*L. fabalis*	ME	46	46	23	33	41°11′54″N	8°42′43″W
27. Madalena	MAD	July 2013; December 2014; June 2015	Allopatric	*L. fabalis*	ME	49	40	16	0	41°06′08″N	8°39′46″W
Total						1,825	1,059	819	702		

a There were both *Fucus* spp. and *Mastocarpus* spp. where individuals were collected but they were essentially found within the first.

b Genotyped by Carvalho et al. (2016).

c Partially genotyped by Carvalho et al. (2016).

d Sequenced by Sotelo et al., 2020).

e Partially sequenced by Sotelo et al., 2020).

f The population of Morás was considered of undetermined distribution given its low sample size and juvenile stage of individuals, precluding the visual assessment of the species present in this location.

g The samples from As Mariñas (18) and from Leça da Palmeira (26) correspond to a group of samples collected in three geographically nearby sites each (<1 km).

### 2.2 Analysis of penis morphology

After carefully removing the soft tissue from the shell, individuals were inspected for the presence of male genitalia. Males (*N* = 818) were initially preclassified into species based on their penis “appearance” (i.e., visual classification). This consisted of comparing the length of the filament with respect to total penis length. A relative length of 10%–25% was considered typical of *L. obtusata* and of 30%–60% typical of *L. fabalis*. Individuals were classified as intermediate when the proportion was 25%–30% or as unknown when they differed substantially from the typical proportions for the two species (Carvalho et al., 2016; Reid, 1996). The penis was then dissected at the base of its insertion and preserved separately from the body, both in 96% ethanol.

A discriminant function analysis (DFA) based on linear measurements of the penis was performed to evaluate the accuracy of this visual classification, using the mass r package v7.3.49 (Venables & Ripley, 2002). For those individuals where we were able to retain an intact penis (*N* = 278), seven morphological features were measured (Appendix S1) under a stereomicroscope (Olympus SZ‐CTV), after replacing the 96% ethanol solution by 60% ethanol to relax the penes. A training set was first selected composed of individuals from allopatric sites (confirmed genetically based on microsatellites, see below): Leça da Palmeira and Madalena for *L. fabalis* (*N* = 24), and Redondela and Viana do Castelo for *L. obtusata* (*N* = 44). The prior for the membership to each species was set to the frequency of individuals from each species in the training set. The DFA was then applied to individuals outside the training set. We set the cutoff of the posterior probability (*Pp* = .99) for the classification of individuals as pure *L. fabalis* or *L. obtusata*, for both the training set and other sites (*N* = 210).

### 2.3 Geometric morphometric analyses of the shell

**Figure 2:**
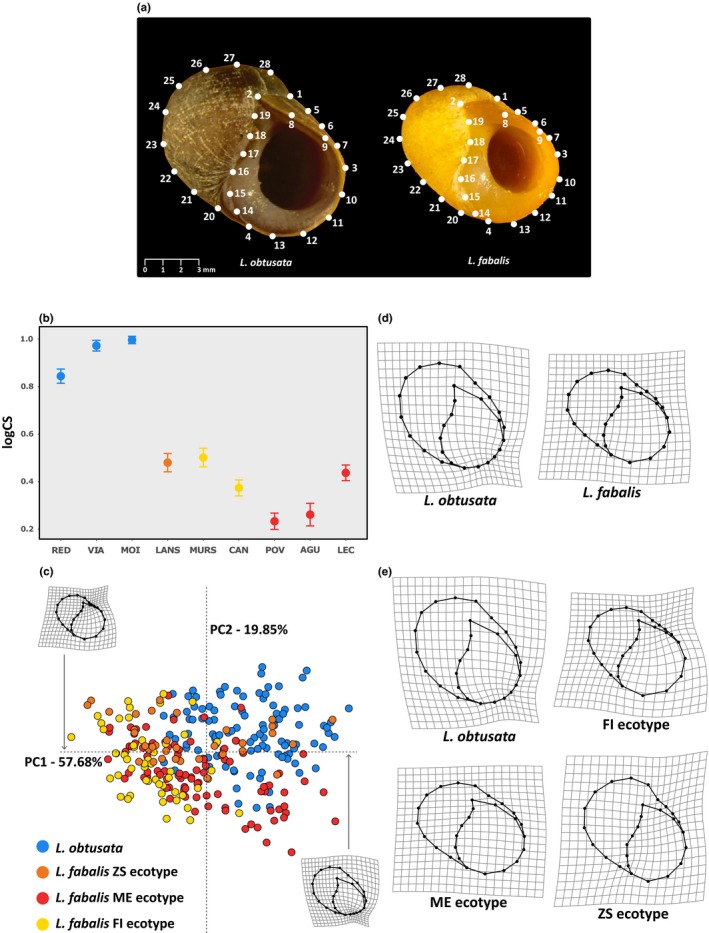
Geometric morphometric (GM) analysis of the shell. (a) Landmarks (LM) digitized in specimens of *Littorina obtusata* (left) and *Littorina fabalis* (FI ecotype; right), placed in the standard position. LM1–LM4 represent fixed LMs, whereas all the other points represent semilandmarks. (b–e) Results for the characterization of shape and size for the allopatric reference populations. (b) Mean log—centroid size (CS) ordered by species and ecotypes (vertical bars denote 95% confidence intervals). Population codes are described in Table 1. (c) Plot of the two first principal components (PC1 and PC2) of shape variation and deformation grids at maximum and minimum PC1 values compared to the global mean. (d, e) Deformation grids depicting the mean shapes for each species and ecotype, respectively. Mean shapes were magnified 2× to enhance visualization

To evaluate variation in shell morphology between *L. fabalis* and *L. obtusata*, identify putative hybrids, and examine the effect of geographical context (i.e., allopatry vs. sympatry) on phenotypic variation, the shells were characterized using landmark‐based geometric morphometrics (GM) (Adams, Rohlf, & Slice, 2013; Dryden & Mardia, 2016). For this purpose, each shell was photographed in a standardized position over graph paper (for scale) using a stereomicroscope (Olympus SZx16) with an attached camera (Olympus SDF PLAPO 1XPF), following the protocol developed for *L. saxatilis* by Carvajal‐Rodriguez et al. (2005). In each shell, we then digitized a total of 28 landmarks (four fixed and 24 sliding semilandmarks, Figure 2a) using tpsdig v1.40 (Rohlf, 2006). While fixed landmarks (LM) represent homologous points of biological interest, semilandmarks describe curves between fixed points, and homology is present in the curves themselves and not in the coordinates’ location (Zelditch, Swiderski, Sheets, & Fink, 2004). Contrary to fixed LM, semilandmarks are treated in a different mathematical manner during superimposition, as they are allowed to slide along the tangent of their position in the curve they define in order to minimize the Procrustes distance among all individuals (Bookstein, 1997). Concerning fixed LMs (Figure 2a), LM1 corresponds to the outer border of the suture; LM2 represents the end of the suture; the position where curves originating at LM1 and LM2 intersect is labeled LM3; and LM4 represents the point where the outer border of the columella starts to develop. The remaining coordinates represent semilandmarks.

Landmark coordinates were superimposed using Generalized Procrustes Analysis (Rohlf & Slice, 1990), to standardize the scale, location, and rotation, and to optimize the position of semilandmarks by minimizing bending energy (Mitteroecker & Gunz, 2009). This provided shape variables (i.e., Procrustes residuals) and centroid size (CS; Dryden & Mardia, 2016), the latter used as an estimate of shell size for GM analyses. This dataset comprised 702 adult individuals, including males and females from all locations except five: As Mariñas, Mougás, and Madalena (ME ecotype), because shell manipulation resulted in their destruction due to their small size; Lanzada North (regarding only the individuals of the *L. fabalis* ZS ecotype); and Morás (*L. obtusata*), because most sampled individuals were juveniles. Nevertheless, samples from these five locations were included in the genetic analyses.

#### 2.3.1 Shell variation across species and ecotypes

To characterize variation in shell morphology across species and ecotypes of *L. fabalis* without the influence of ongoing hybridization, we first conducted a set of analyses on individuals from allopatric populations (*L. obtusata*, *N* = 108; *L. fabalis*, *N* = 174; Figure 1, Table 1). We used a principal component analysis (PCA) of shape variables to explore general patterns of shape variation. To test whether *L. obtusata* and the three ecotypes of *L. fabalis* differed in shell size and shape, we examined general linear models (GLM) for logCS and the multivariate set of shape variables in two sequential analyses: the first with species (grouping all *L. fabalis* ecotypes) as the main factor and location of collection as a factor nested within species; and the second within *L. fabalis*, with ecotype as the main factor and location also as a nested factor. To account for possible allometric effects of size on shape, we also examined a GLM for shape using size as a covariate, with the same main factors as before, and location as a nested factor. The significance of different terms was evaluated using residual randomization in permutation procedures based on Procrustes distances and consisting of 1,000 permutations, as implemented in the function *procD.lm* of *geomorph* R package (Adams, Collyer, & Kaliontzopoulou, 2018), and *Z*‐scores were used for significance testing, as recommended by Adams and Collyer (2016). Shape differences between groups (species and ecotypes of *L. fabalis*) were visualized using deformation grids.

#### 2.3.2 Hybridization effects on shell morphology

In order to identify shell morphological features that differ the most between the two species and examine whether genetic hybrids exhibited intermediate shell morphology, we performed a DFA on shape using the r package mass v7.3.50. The DFA was constructed using individuals from reference allopatric populations of both species (*N* = 282), including all *L. fabalis* ecotypes, aiming to capture as much morphological variation as possible. The *Pp* of assignment of each individual to each species based on shell shape was estimated using a leave‐one‐out cross‐validation procedure. Based on this discriminant function, we then inferred the morphological *Pp* of each individual from the remaining populations (from sympatric sites) and for which a genetic assignment was available (*N* = 339), in order to compare the morphological predicted shapes with the genetic membership coefficients obtained with structure (global analysis, total *N* = 556; see below).

#### 2.3.3 Species differences in shell morphology across different geographic contexts

To examine how the contact between species may influence shell shape and size irrespective of hybridization, we compared the overall differences between the two species in distinct geographic contexts (allopatry vs. sympatry). As we were interested in examining morphological variation of the genetically pure individuals of each species, samples for which no genetic information was available and those genetically classified as hybrids in the global structure analysis were removed from the dataset. This resulted in a new subset of 540 individuals: 117 *L. fabalis* and 100 *L. obtusata* from allopatric populations and 183 *L. fabalis* and 140 *L. obtusata* from sympatric populations. A GLM analysis was implemented to examine how shell morphology responds to species sympatry. We sequentially examined shell size, shape, and shape while taking size variation into account and fitted GLMs that included geographical context and species, as well as their interaction. As in previous analyses, we also included sampling location as a nested factor to account for local variation among populations.

### 2.4 Molecular methods

The genetic characterization of the individuals collected during this study was based on two types of markers: microsatellites and mtDNA. While microsatellites were shown to distinguish the two species and identify recent‐generation hybrids (Carvalho et al., 2015, 2016), mtDNA demonstrated introgressive hybridization between flat periwinkles (Kemppainen et al., 2009; Sotelo et al., 2020).

#### 2.4.1 DNA extraction

Genomic DNA was extracted from head–foot tissue using a modified version of the standard high‐salt protocol (Sambrook, Maniatis, & Fritsch, 1989), by replacing the lysis buffer by cetyltrimethyl ammonium bromide (Winnepenninckx, Backeljau, & Wachter, 1993). The final DNA concentration was standardized across samples (~5 to 10 ng/μl).

#### 2.4.2 Microsatellite genotyping

From the battery of 17 microsatellite loci developed for *L. fabalis* (Carvalho et al., 2015), 12 markers (four of each di‐, tri‐, and tetra‐nucleotides repeat motifs) were selected based on their informativeness for species discrimination, as well as on their genotyping reliability after combining them into two multiplexes (Appendix S2). Amplification reactions were performed as in Carvalho et al. (2016) and run on a ABI 3730 sequencer (Applied Biosystems) at STABVIDA. Genotyping was carried out using PeakScanner v1.0 (Applied Biosystems) but later manually inspected. To evaluate the consistency of the obtained genotypes, at least 10% of the samples were amplified and genotyped twice. From the 1,059 samples analyzed in this study, 344 (32.5%) had previously been genotyped for microsatellites by Carvalho et al. (2016; Table 1). To rule out any potential discrepancies in allele scoring between the two sample sets, new amplification and genotyping were performed for 5% of the samples from Carvalho et al. (2016).

#### 2.4.3 Mitochondrial DNA amplification and sequencing

A fragment of the mitochondrial gene cytochrome b (*Cyt‐b*) was amplified using the cytbF‐cytbR primer pair (Panova et al., 2011). Amplification was carried out as in Sotelo et al., 2020 and Sanger sequencing was performed at Macrogen Europe (Amsterdam, The Netherlands) using the forward primer. Chromatograms were visually inspected and sequences aligned using Sequencher 4.1.4 (Gene Codes Corporation), with subsequent trimming for equal length (569 bp) and manual correction of artifacts. From the 819 samples analyzed in this study, 117 (14.3%) had previously been sequenced for this fragment by Sotelo et al., 2020 Table 1).

### 2.5 Genetic data analyses

#### 2.5.1 Hybridization between *L. fabalis* and *L. obtusata* based on microsatellites

The extent and patterns of hybridization between these sister species were investigated with structure v2.3.4 (Falush, Stephens, & Pritchard, 2003, 2007; Hubisz, Falush, Stephens, & Pritchard, 2009; Pritchard, Stephens, & Donnelly, 2000) and newhybrids v1.1 (Anderson & Thompson, 2002) based on 11 loci that passed quality control filters for Hardy–Weinberg and linkage disequilibria (Appendix S2). structure runs were performed at two different scales: global and local. At the global scale, the multilocus genotypes of the entire dataset were given as input to structure. The extent of hybridization was also evaluated at a local scale since, in low‐dispersal species like flat periwinkles, hybridization will likely occur in an independent manner in each location. All structure analyses were based on five replicates with 1,000,000 MCMC iterations after a burn‐in of 100,000 steps, and under an admixture model with independent allele frequencies, setting *k* = 2. In order to define the threshold of *Q* (hereafter T*Q*) used to classify individuals as pure or hybrid, we implemented a slightly modified version of the method from Hasselman et al. (2014) that minimizes misclassifications based on simulations with hybridlab v1.0 (Nielsen, Bach, & Kotlicki, 2006; Appendix S2).

Finally, a more detailed classification of hybrids was performed with newhybrids, which retrieves a *Pp* of the membership of each individual to the different genotype classes: F, parental *L. fabalis*; O, parental *L. obtusata*; F1, offspring of a cross between F and O; F2, offspring of a cross between F1s; BCF, offspring of an F1 backcrossed with parental *L. fabalis*; and BCO, offspring of an F1 backcrossed with parental *L. obtusata*. Taking into account the structure results, newhybrids analyses were only performed for the global dataset without a priori information on allele frequencies or admixture proportions. We used a “Jeffrey's‐like” prior, which considers that some alleles may be rare or absent in the different populations and so more accurately determines the assignment of hybrid individuals to their respective categories (Anderson, 2003). To assess the consistency of estimates, three replicates of 1,000,000 MCMC iterations, after a burn‐in of 100,000, were performed. A procedure to estimate the threshold of *Pp* (*TPp*) for the classification of individuals as pure or hybrid, similar to the one applied for *TQ* (structure), was implemented (Appendix S2).

#### 2.5.2 MtDNA introgression

The number of haplotypes was assessed using DnaSP v6.11.01 (Rozas et al., 2017). The relationships between haplotypes were inspected by constructing a network using tcs v1.21 (Clement, Posada, & Crandall, 2000) under default parameters (95% connection limit criterion), which was later edited with TcsBU (Santos et al., 2016). MtDNA introgression (globally and for each sampling site) was estimated as the proportion of individuals from each genotypic group defined by the global structure analysis (*L. fabalis* and *L. obtusata*) carrying a mtDNA haplotype from the less frequent clade in that group. The percentage of haplotypes from each clade was also estimated for each hybrid category defined by newhybrids.

## 3 RESULTS

### 3.1 Classification of males based on genital morphology

The males analyzed both in terms of visual appearance and using the DFA (based on seven morphological features, with a *Pp* > .99) were classified into species with high concordance (94.3%, excluding 68 individuals from allopatric sites used as reference; Table 2). The differences correspond to individuals classified as intermediate using one approach and as pure of one species using the other approach and with a single exception were all observed in sympatric sites. The discriminant axis contrasted the length of the filament to the length of the gland row, the number of rows, and the number of glands (Appendix S1). Since the number of samples visually classified was much higher than those available for the DFA, for which only intact penis could be used, the former was used in subsequent analyses.

**Table 2:** Sample size (*N*) and composition of each location in terms of sex: *N* ♀, number of females; *N* ♂, number of males and their corresponding species based on male genitalia using the visual appearance and the discriminant function analysis (DFA); *N* Intermediate, number of males with intermediate genitalia morphology; and *N* Unknown, number of males for which classification was not possible

Location	*N*	*N* ♀	*N* ♂	Visual appearance				DFA assignment		
				*N* ♂ *Littorina fabalis*	*N* ♂ *Littorina obtusata*	*N* Intermediate	*N* Unknown	*N* ♂ *L. fabalis*	*N* ♂ *L. obtusata*	*N* Intermediate
Burela	40	16	18	17	1			14	0	1
Morás	15	6	9	0	9			0	7	1
Abelleira	84	46	38	36	2			—	—	—
Muros North	40	25	15	13	2			—	—	—
Muros South	25	13	12	12	0			—	—	—
Lanzada North	95	67	28	14	14			13	14	0
Lanzada South	78	44	34	34	0			—	—	—
Seixiños	79	46	33	18	14		1	5	2	1
Aldán North	46	22	24	0	22		2	0	22	0
Aldán South	20	9	11	7	4			6	4	0
Borna	66	26	40	29	11			10	10	2
Tirán	147	73	74	73	1			8	1	0
Cangas	150	80	70	52	18			—	—	—
Redondela	57	23	34	0	34			0	18	0
La Guia	62	25	37	23	14			11	14	0
Alcabre	53	32	21	11	10			6	10	1
Canido	93	44	49	49	0			—	—	—
As Mariñas	74	42	32	32	0			—	—	—
Mougás	24	12	12	12	0			—	—	—
Viana do Castelo	60	33	27	0	27			0	26	0
Rio de Moinhos	85	43	42	0	42			—	—	—
Póvoa de Varzim	63	40	23	23	0			—	—	—
Mindelo	90	62	28	15	13			11	11	1
Agudela	65	37	28	28	0			—	—	—
Cabo do Mundo	125	75	50	13	30	6	1	11	12	1
Leça da Palmeira	46	28	18	18	0			17	0	0
Madalena	49	38	11	11	0			7	0	0

#### 3.1.1 Variation in shell morphology

The GM analysis provided 52 shape variables plus CS. The two species differed significantly (*Z* = 1.511, *p* = .009) in terms of size, with *L. obtusata* being larger than *L. fabalis* (Figure 2b, Table 3), despite significant variation among locations (*Z* = 6.460, *p* = .001). Within *L. fabalis*, significant variation in CS was observed among sampling locations (*Z* = 3.736, *p* = .001), but no significant differences were observed among ecotypes (Table 3). The PCA of shape variables retrieved two components that cumulatively explained over 75% of total shape variation (Figure 2c). The two sister species occupy different parts of this morphospace despite some overlap, while the distinction among *L. fabalis* ecotypes was less evident, as expected. Accordingly, GLMs revealed significant shape differences both between species and among ecotypes of *L. fabalis*, despite significant local variation (Table 3). Differences in shape among locations and ecotypes of *L. fabalis* were also significant irrespective of size variation (*Z* = 2.039, *p* = .016; and *Z* = 2.604, *p* = .006, respectively), but this was not the case between species (Table 3). Indeed, location was the factor with the strongest effect on shape variation, followed by species and then ecotype (as captured by Z‐scores, Collyer & Adams, 2013; Collyer, Sekora, & Adams, 2015). The examination of deformation grids indicated that the shell of *L. obtusata* is overall more elongated and tends to have a relatively smaller aperture than *L. fabalis*, which tends to have a shell with rounder shape and wider aperture (Figure 2d). Although less pronounced, differences among ecotypes of *L. fabalis* included a reduction of the suture border and a widening of the aperture in the FI ecotype, while the ZS ecotype exhibited the opposite tendency, resembling the shape of *L. obtusata* (Figure 2e).

**Table 3:** General linear model analysis (GLMs) of morphological differences in terms of size and shape, as well as shape accounting for the influence of size (CS)

GLMs				
	*df*	*SS*	*Z*	*p*
Centroid Size				
*Littorina fabalis* versus *Littorina obtusata*				
Species	1	20.636	1.511	.009*
Location	7	2.182	6.460	.001*
Residuals	273	2.354		
Total	281	25.171		
*L. fabalis* ecotypes				
Ecotype	2	0.747	0.139	.447
Location	3	0.952	3.736	.001*
Residuals	168	1.814		
Total	173	3.513		
Shape				
*L. fabalis* versus *L. obtusata*				
Species	1	0.757	2.700	.003*
Location	7	0.544	6.608	.001*
Residuals	273	2.414		
Total	281	3.715		
*L. fabalis* ecotypes				
Ecotype	2	0.339	1.877	.030*
Location	3	0.105	2.863	.002*
Residuals	168	1.563		
Total	173	2.007		
Shape accounting for size				
*L. fabalis* versus *L. obtusata*				
CS	1	0.7830	6.054	.001*
Species	1	0.0334	−0.782	.789
Location	7	0.5478	6.683	.001*
Residuals	272	2.3507		
Total	281	3.7148		
*L. fabalis* ecotypes				
CS	1	0.0379	2.200	.014*
Ecotype	2	0.3968	2.604	.006*
Location	3	0.0673	2.039	.016*
Residuals	167	1.5052		
Total	173	2.0072		

Note

Only individuals from allopatric sites were included in this analysis.

Abbreviations: *df*, degrees of freedom; *SS*, sums of squares; *Z*, Z‐scores*; p*, *p*‐value (*indicates significant values).

#### 3.1.2 *Morphological variation in allopatric *versus* sympatric populations*

**Figure 3:**
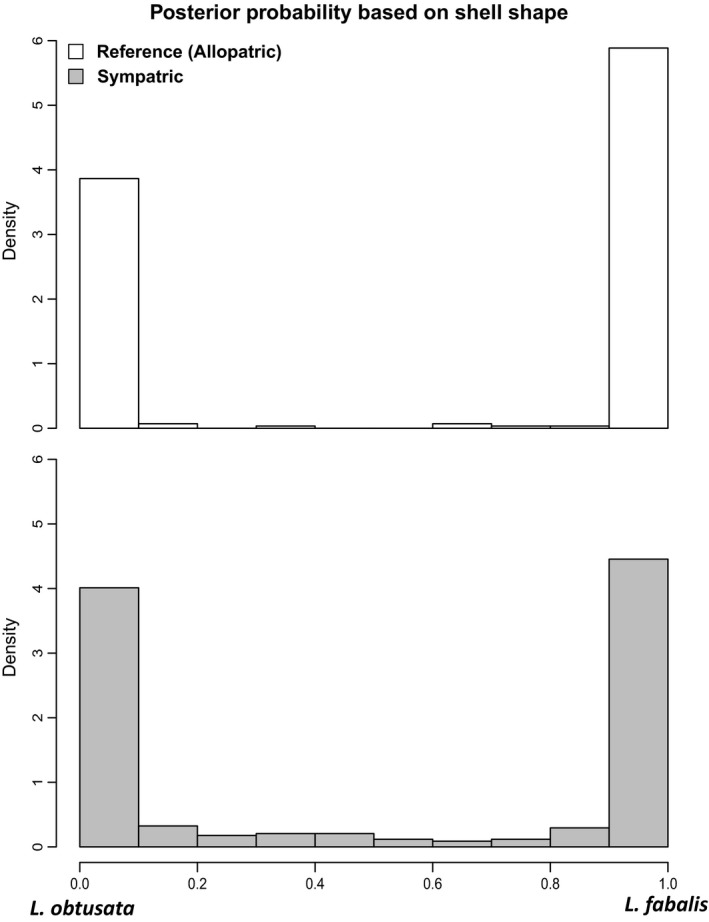
Posterior probability (*Pp*) distribution of morphological assignments based on the discriminant function analysis (DFA) constructed from allopatric populations (top) and used to classify individuals from sympatric populations (bottom). In both graphs, *Pp* values represent assignment probability to *Littorina fabalis*

The DFA of allopatric populations rendered a high percentage (97.35%) of correct assignments of reference individuals to species (274 out of 282) based solely on shell shape morphology. By contrast, the percentage of individuals confidently assigned to each species (i.e., with a *Pp* ≥ .90) was lower for the sympatric populations (356 out of 420 individuals, 84.8%), where 64 individuals with intermediate shell morphologies were identified (Figure 3, Table 4).

**Table 4:** Classification of individuals for each location based on a geometric morphometric (GM) analysis of the shell

Location	*N* GM	GM ≥ .90 *Littorina fabalis*	GM ≥ .90 *Littorina obtusata*	Intermediate shape
Burela	20	18	1	1
Abelleira	31	13	11	7
Muros North	14	7	5	2
Muros South^a^	19	18	0	1
Lanzada North	30	3	22	5
Lanzada South^a^	34	32	0	2
Seixiños	38	23	7	8
Aldán North	23	4	7	12
Aldán South	8	2	5	1
Borna	24	6	13	5
Tirán	36	30	3	3
Cangas	49	17	24	8
Redondela^a^	36	0	35	1
La Guia	32	5	22	5
Alcabre	29	2	23	4
Canido^a^	34	34	0	0
Viana do Castelo^a^	36	0	36	0
Rio de Moinhos^a^	36	0	33	3
Póvoa de Varzim^a^	28	28	0	0
Mindelo	47	33	14	0
Agudela^a^	26	26	0	0
Cabo do Mundo	39	28	8	3
Leça da Palmeira^a^	33	32	0	1
Total	702	361	269	72

Note

*N* GM, total number of individuals analyzed; GM ≥ .90, number of individuals with a posterior probability equal to or higher than .90 to either species; Intermediate shape, number of individuals not classified to species (posterior probability below .90 to both of them).

a Allopatric sites were analyzed separately from the sympatric ones (see Section 2).

**Figure 4:**
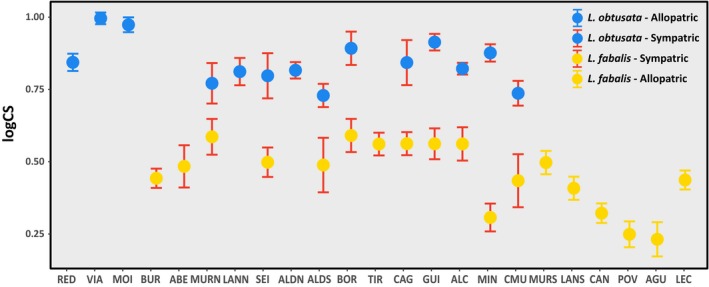
Shell size of *Littorina obtusata* and *Littorina fabalis* allopatric and sympatric populations represented by mean centroid size (CS), with vertical bars denoting 95% confidence intervals. Population codes are described in Table 1

After excluding hybrids, and despite significant local variation as represented by sampling location, species and geographic context (i.e., allopatric vs. sympatric) interacted significantly in their effect on size (*Z* = 1.572, *p* = .005), but not on shape (Figure 4, Table 5). Species and geographic context also did not interact significantly when taking size variation into account (Table 5). The examination of CS variation across locations indicated that the two species tend to be more similar in size in sympatry, as *L. obtusata* becomes slightly smaller and *L. fabalis* slightly larger than in allopatry (Figure 4).

**Table 5:** General linear model analysis (GLMs) of shape and size variation between species taking into account the effect of the geographical context of divergence (Geog) and sample location as a nested factor

GLMs				
	*df*	*SS*	*Z*	*p*
Centroid Size				
Species	1	24.215	2.111	.001*
Geog	1	0.070	0.169	.505
Species:Geog	1	1.787	1.572	.005*
Location	20	2.791	7.696	.001*
Residuals	516	5.307		
Total	539	34.171		
Shape				
Species	1	0.931	3.156	.001*
Geog	1	0.211	1.382	.091
Species:Geog	1	0.092	0.401	.340
Location	20	1.691	11.199	.001*
Residuals	516	4.251		
Total	539	7.176		
Shape accounting for size				
CS	1	0.914	6.221	.001*
Species	1	0.170	1.200	.130
Geog	1	0.221	1.440	.084
Species:Geog	1	0.041	−0.580	.718
Location	20	1.682	11.074	.001*
Residuals	515	4.148		
Total	539	7.176		

Note

Individuals genetically classified as hybrids were excluded from this analysis.

Abbreviations: *df*, degrees of freedom; *SS*, sums of squares; *Z*, Z‐scores; *p*, *p*‐value (*indicates significant values).

#### 3.1.3 Classification of individuals using genetics and assessment of hybridization

**Figure 5:**
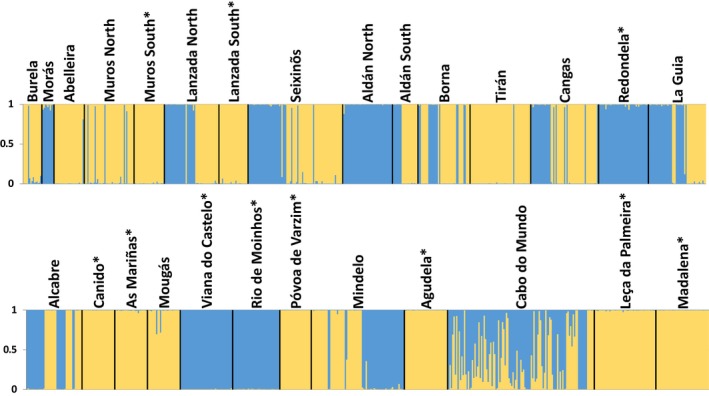
Membership coefficient (*Q* ranging from 0 to 1) of all genotyped individuals to the clusters identified by the initial structure analysis for *k* = 2. Each vertical bar represents an individual, where typical *Littorina fabalis* ancestry is represented in yellow and typical *Littorina obtusata* ancestry in blue. Vertical bars with both yellow and blue represent individuals with admixed ancestry. On top, location names follow Table 1 and “*” indicates allopatric locations. Results are consistent across replicates

The initial structure analysis revealed a clear distinction between the two species (Figure 5). For the global‐scale analysis, the threshold of *Q* (*TQ*) that minimized the number of misclassifications between hybrid and pure individuals corresponded to 0.90, with 97.75% of simulated genotypes being correctly classified as pure or admixed (Appendix S3). At the local scale, *TQ* values ranged from 0.88 in Mougás + Redondela and Abelleira + Muros North to 0.94 in Cabo do Mundo and Lanzada North (Appendix S4).

The structure global analysis of the empirical dataset revealed 61 hybrids distributed over seven locations, with hybridization ranging from 2.1% in La Guia to 46.9% in Cabo do Mundo (Table 6). The local analyses revealed a lower number of locations with hybrids (three) but a higher proportion of hybrids within Cabo do Mundo (56.8%; Table 6). Importantly, hybrids were consistently found in Mougás, Mindelo, and Cabo do Mundo, independently of the scale.

**Table 6:** Number of hybrids between *Littorina fabalis* and *Littorina obtusata* detected across locations using structure (left) and newhybrids (right)

Location	*N* Analyzed	structure		newhybrids		
		Global analysis	Local analyses	*N* Assigned	Hybrids in each hybrid class	Hybrid class
		*N* (%)	*N* (%)	(*Pp* ≥ .80)	*N* (%)	(*N*)
Burela	15	0	0	15	0	
Morás	9	0	0	8	0	
Abelleira	24	1 (4.17)	0	23	0	
Muros North	40	0	0	38	0	
Muros South	24	0	0	24	0	
Lanzada North	44	0	0	43	0	
Lanzada South	23	0	0	23	0	
Seixiños	77	2 (2.60)	0	74	0	
Aldán North	40	1 (2.50)	0	40	0	
Aldán South	20	0	0	20	0	
Borna	42	0	0	41	0	
Tirán	49	0	0	49	0	
Cangas	55	0	0	54	0	
Redondela	40	0	0	37	0	
La Guia	47	1 (2.13)	0	47	0	
Alcabre	42	0	0	42	0	
Canido	24	0	0	24	0	
As Mariñas	24	0	0	24	0	
Mougás	24	2 (8.33)	1 (4.17)	24	2 (8.33)	F2 (2)
Viana do Castelo	39	0	0	39	0	
Rio de Moinhos	35	0	0	35	0	
Póvoa de Varzim	23	0	0	23	0	
Mindelo	70	2 (2.86)	2 (2.86)	70	3 (4.29)	BCO (3)
Agudela	32	0	0	32	0	
Cabo do Mundo	111	52 (46.85)	63 (56.76)	77	27 (35.06)	F2 (3), BCO (18), BCF (6)
Leça da Palmeira	46	0	0	46	0	
Madalena	40	0	0	40	0	
Total	1,059	61	66	1,012	32	

Note

*N* (Analyzed) is the number of individuals analyzed with both software. For the Global analysis (structure), the entire dataset was used as a single input; for the Local analyses, multiple inputs were used (by location or joining closest locations when required, see Section 2). For newhybrids, *N* Assigned, number of individuals analyzed that were classified to any class. The class of the identified hybrids is also indicated by location, and the number of individuals per class is shown between brackets (F—*L. fabalis*, O—*L. obtusata*, F1 and F2 hybrids, and backcrosses to each parental class—BCF and BCO). The percentages were calculated in respect to the sample size (*N* Analyzed) for each location.

The newhybrids analyses revealed a similar pattern, assigning most simulated genotypes to their predefined class, and, more importantly, no parental genotypes were assigned to hybrid classes. The threshold of posterior probability (*TPp* = .80) allowed the correct classification of over 85% of individuals, with low underlying error rate (0.4%). Based on this threshold, 97.5% of simulated *L. obtusata*, 98% of simulated *L. fabalis*, and 83.3% of hybrid genotypes were correctly assigned, with 78% of hybrid genotypes assigned to the correct hybrid class.

Our simulations show that under the current settings both structure and newhybrids erroneously assigned more hybrid genotypes as pure than vice versa, suggesting that our estimates of the number of hybrids are conservative.

Concerning the real dataset, 1,012 from the 1,059 analyzed individuals were unambiguously assigned to genotype classes, whereas the remaining individuals (4% of the total) could not be assigned with confidence to any of the six classes (*Pp* ≥ .80). Among the confidently assigned individuals, only 32 were classified as hybrids by newhybrids, distributed over three locations (Table 6). No individual was classified as F1. Both hybrids from Mougás were classified as F2 and the three hybrids from Mindelo as BCO. Most of the hybrids detected in Cabo do Mundo were classified as BCO (18 individuals), while six were classified as BCF and three as F2 hybrids. Finally, among the 17 individuals from Cabo do Mundo assigned to a hybrid class with *Pp* < .80, five had highest probability of assignment to BCO, five to BCF, three to F2, and four to F1. Additionally, one individual from Lanzada North was assigned to BCF with *Pp* < .80, making a total of 50 hybrids over all locations. Comparing the two approaches (global structure and newhybrids) for hybrid identification, the same two individuals from Mindelo, two from Mougás, and 43 individuals from Cabo do Mundo were always detected as hybrids, independently of the approach used.

**Figure 6:**
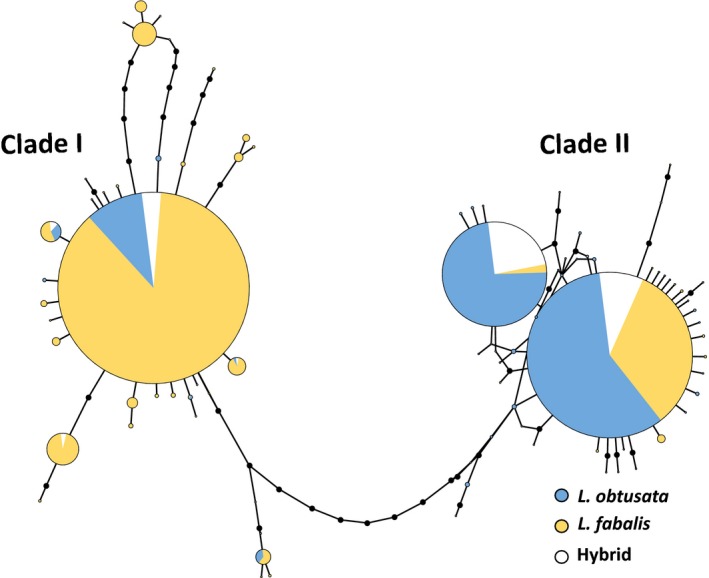
Network of the mtDNA haplotypes, built with TCS (under a 95% connection limit criterion) and modified with TcsBU. Black dots represent missing haplotypes, whereas lines between dots/circles represent mutations. Individuals were classified as *Littorina fabalis*, *Littorina obtusata*, or hybrids based on the global structure analysis of microsatellite data

Two main clades of mtDNA haplotypes were identified (Figure 6). From a total of 762 sequenced individuals that were also analyzed for microsatellites, 423 carried haplotypes from clade I (typical of *L. fabalis*) and 339 presented haplotypes from clade II (typical of *L.* *obtusata*) (Table 7). The total proportion of *L. obtusata‐*typical haplotypes present in *L. fabalis* individuals was slightly higher than the other way around (18.5% and 16.2%, respectively). However, the proportion of atypical haplotypes was very heterogeneous among sites (Table 7). Thirteen out of the 22 *L. fabalis* sites sequenced for mtDNA showed typical *L. obtusata* haplotypes, with the proportion varying between 7.1% (Alcabre) and 91.7% (Aldán South). Among *L. obtusata* sites, eight out of 17 showed typical *L. fabalis* haplotypes, with the proportion varying from 14.3% (Alcabre) to 100% (Burela; Table 7). Finally, the proportion of sites with atypical haplotypes in either species was higher in sympatric than allopatric sites (84.6% vs. 30.8%).

**Table 7:** Mitochondrial DNA introgression between flat periwinkles in each location

Location	Ecotype	*N*	*N Littorina fabalis*	*N Littorina obtusata*	Clade I	Clade II	Introgression into *L. fabalis* (%)	Introgression into *L. obtusata* (%)
Burela	ME/FI^a^	15	14	1	15	0	0/14 (0)	1/1 (100)
Morás	—	9	0	9	6	3	—	6/9 (66.67)
Abelleira	FI	17	17	0	15	2	2/17 (11.76)	—
Muros North	FI	35	30	5	30	5	0/30 (0)	0/5 (0)
Muros South^b^	FI	15	15	0	15	0	0/15 (0)	—
Lanzada North	ZS	39	19	20	9	30	10/19 (52.63)	0/20 (0)
Lanzada South^b^	ZS	22	22	0	15	7	7/22 (31.82)	—
Seixiños	ZS	49	25	24	13	36	12/25 (48.00)	0/24 (0)
Aldán North^b^	—	19	0	19	0	19	—	0/19 (0)
Aldán South	FI	18	12	6	1	17	11/12 (91.67)	0/6 (0)
Borna	FI	42	27	15	23	19	9/27 (33.33)	5/15 (33.33)
Tirán	FI	40	39	1	35	5	4/39 (10.26)	0/1 (0)
Cangas	FI	41	22	19	27	14	4/22 (18.18)	9/19 (47.37)
Redondela^b^	—	23	0	23	5	18	—	5/23 (21.74)
La Guia	FI	36	13	23	20	16	2/13 (15.38)	9/23 (39.13)
Alcabre	FI	35	14	21	16	19	1/14 (7.14)	3/21 (14.29)
Canido^b^	FI	21	21	0	21	0	0/21 (0)	—
As Mariñas^b^	ME	19	19	0	12	7	7/19 (36.84)	—
Mougás^b^	ME	18	18	0	9	9	9/18 (50.00)	—
Viana do Castelo^b^	—	20	0	20	0	20	—	0/20 (0)
Rio de Moinhos^b^	—	30	0	30	0	30	—	0/30 (0)
Póvoa de Varzim^b^	ME	21	21	0	21	0	0/21 (0)	—
Mindelo	ME	66	34	32	34	32	0/34 (0)	0/32 (0)
Agudela^b^	ME	19	19	0	19	0	0/19 (0)	—
Cabo do Mundo	ME/FI^a^	54	19	35	23	31	7/19 (36.84)	11/35 (31.43)
Leça da Palmeira^b^	ME	23	23	0	23	0	0/23 (0)	—
Madalena^b^	ME	16	16	0	16	0	0/16 (0)	—
Total		762	459	303	423	339	85/459 (18.51)	49/303 (16.17)

Abbreviations: *N*, number of samples sequenced for mtDNA; *N L. fabalis*, number of individuals classified as *L. fabalis* based on structure global analysis that were sequenced for mtDNA; *N L. obtusata*, number of individuals classified as *L. obtusata* based on structure global analysis that were sequenced for mtDNA; Clade I, number of individuals with mtDNA haplotype from clade I (typical of *L. fabalis*); Clade II, number of individuals with mtDNA haplotype from clade II (typical of *L. obtusata*); introgression into *L. fabalis*, number (and percentage) of haplotypes from clade II over the number of individuals classified as *L. fabalis* based on structure; and introgression into *L. obtusata*, number (and percentage) of haplotypes from clade I over the number of individuals classified as *L. obtusata* based on structure.

a Individuals were found in *Fucus* spp. but the co‐occurrence of *Mastocarpus* spp. does not allow an accurate classification of the ecotype.

b Allopatric sites based on field observations during sampling.

Among the individuals classified as hybrids using structure that were also sequenced for mtDNA (*N* = 57), 77.2% carried a typical *L. obtusata* haplotype (clade II) (Table 8, Figure 6), whereas the remainder carried a typical *L. fabalis* haplotype. Nevertheless, these proportions are mainly driven by Cabo do Mundo, where most hybrids were detected. All detected hybrid classes showed a higher percentage of *L. obtusata* mtDNA haplotypes (81.5% in total; Table 8).

**Table 8:** Mitochondrial DNA clades of the hybrids identified with structure in each location and *Littorina fabalis* ecotype involved, and the same relatively to the hybrids identified with NEWHYRBIDS for each class

	*Littorina* *fabalis* ecotype	*N*	Clade I (typical *L. fabalis*)	Clade II (typical *Littorina obtusata*)
Location				
Abelleira	FI	1	0	1
Lanzada North	ZS	1	0	1
Seixiños	ZS	2	2	0
Cangas	FI	1	0	1
La Guia	FI	1	1	0
Mougás	ME	2	0	2
Mindelo	ME	2	1	1
Cabo do Mundo	a	47	9	38
Total		57	13	44
Hybrid class				
F2	ME/a	5	0	5
BCO	ME/a	17	4	13
BCF	ME/a	5	1	4
Total		27	5	22

Abbreviations: *N,* total number of hybrids according to each software sequenced for mtDNA; Clade I, number of hybrids with mtDNA haplotype from clade I (typical of *L. fabalis*); Clade II, number of hybrids with mtDNA haplotype from clade II (typical of *L. obtusata*).

a It was not possible to clearly distinguish between ME and FI.

#### 3.1.4 Comparison of classification based on genetics, male genitalia, and shell morphology

None of the individuals assigned to one species by the structure analysis was classified into the other species by either approach based on the genital morphology (*N* = 565, 337 *L. fabalis* and 228 *L. obtusata*). The converse was also true: genital classification to parental groups was 100% congruent with structure‐based classification. However, only five individuals were simultaneously classified as intermediate based on male genitalia (visual inspection or DFA) and genetics (microsatellites) out of 31 individuals that were intermediate in one classification or the other.

The concordance was lower for individuals analyzed for both shell morphology and microsatellites (*N* = 556), where only 424 out of the 540 genetically pure individuals (79% of *L. fabalis* and 77.9% of *L. obtusata*) were morphologically assigned to their corresponding species with *Pp* ≥ .90 (Table 9). Several genetically pure individuals (*N* = 61) exhibited intermediate shell shape (Table 9), most of them (*N* = 53) were from sympatric sites. In contrast, none of the genetically identified hybrids that were also morphologically analyzed (*N* = 16) presented intermediate shell shape.

**Table 9:** Comparison between the classification of individuals based on genetics (microsatellites) and shell morphology for each location

Location	*N* GEN and GM data	GEN and GM	GEN and GM	Intermediate shape	GEN and GM	GEN Hybrids
		*Littorina fabalis*	*Littorina obtusata*		Mismatch	GM assigned to species
		*N* (%)	*N* (%)	*N* (%)	*N* (%)	*N* (species)
Burela	12	11 (91.67)	—	0	1 (8.33)	
Abelleira	16	6 (40.00)	—	5 (33.33)	4 (26.67)	1 (*L. obtusata*)
Muros North	14	6 (60.00)	2 (50.00)	2 (14.29)	4 (28.57)	
Muros South^a^	18	17 (94.44)	—	1 (5.56)	0	
Lanzada North	19	—	14 (73.68)	3 (15.79)	2 (10.53)	
Lanzada South^a^	14	12 (85.71)	—	2 (14.29)	0	
Seixiños	37	16 (64.00)	3 (27.27)	8 (22.22)	9 (25.00)	1 (*L. fabalis*)
Aldán North	22	—	6 (28.57)	11 (52.38)	4 (19.05)	1 (*L. obtusata*)
Aldán South	8	2 (50.00)	4 (100)	1 (12.50)	1 (12.50)	
Borna	21	4 (26.67)	4 (66.67)	5 (23.81)	8 (38.10)	
Tirán	32	26 (81.25)	—	3 (9.38)	3 (9.38)	
Cangas	25	10 (62.50)	6 (66.67)	4 (16.00)	5 (20.00)	
Redondela^a^	36	—	35 (97.22)	1 (2.78)	0	
La Guia	32	4 (33.33)	17 (85.00)	5 (15.63)	6 (18.76)	
Alcabre	22	2 (40.00)	16 (94.12)	3 (13.64)	1 (4.55)	
Canido^a^	18	18 (100)	—	0	0	
Viana do Castelo^a^	36	—	36 (100)	0	0	
Rio de Moinhos^a^	28	—	25 (89.29)	3 (10.71)	0	
Póvoa de Varzim^a^	16	16 (100)	—	0	0	
Mindelo	42	26 (100)	13 (86.67)	0	2 (4.88)	1 (*L. obtusata*)
Agudela^a^	18	18 (100)	—	0	0	
Cabo do Mundo	37	11 (100)	6 (42.86)	3 (12.00)	5 (20.00)	2 (*L. obtusata*); 10 (*L. fabalis*)
Leça da Palmeira^a^	33	32 (96.97)	—	1 (3.03)	0	
Total	556	237	187	61	55	16

Note

Shown is: *N* GEN and GM data, the number of individuals analyzed for both genetics and shell morphology; GEN and GM *L. fabalis N* (%), number (and percentage) of concordant assignment using genetics and shell morphology to *L. fabalis*; GEN and GM *L. obtusata N* (%), number (and percentage) of concordant assignment using genetics and shell morphology to *L. obtusata*; Intermediate shape *N* (%), number (and percentage) of individuals genetically pure with intermediate shell shape; GEN and GM Mismatch *N* (%), number (and percentage) of individuals genetically classified into one species with shell shape typical from the other species; GEN Hybrids, number of individuals genetically hybrid classified as pure from each species based on shell morphology.

a For the GM analysis, allopatric sites were analyzed separately from the sympatric ones (see Section 2).

## 4 DISCUSSION

Characterizing the patterns of hybridization between closely related species pairs is an important step toward understanding the role of gene flow in speciation (Abbott et al., 2013). Introgressive hybridization between flat periwinkles was previously suggested based on mtDNA analyses (Kemppainen et al., 2009), together with the detection of hybrids between the two species in Cabo do Mundo using microsatellites (Carvalho et al., 2016). However, no systematic characterization of hybridization across multiple populations in different geographic contexts has been performed. Therefore, this study has advanced our understanding about the role of gene flow in this system by: (a) integrating genetic analyses (both nuclear and mitochondrial markers) with geometric and linear morphometric tools to characterize shell and male genital morphology, respectively; (b) substantially extending both the number of individuals and of populations analyzed, from both species, providing a comprehensive characterization of hybridization patterns between two species of flat periwinkles across 27 locations in north‐western Iberia; (c) performing a systematic comparison of hybridization patterns between allopatric and sympatric sites; and (d) examining the contributions of *L. fabalis* ecotypes to hybridization between the two sister species.

### 4.1 Morphological and genetic differences and hybrid identification

The concordance found between genetic data and adult males’ genital shape confirms that the latter is a reliable trait to distinguish the two species. On the other hand, the landmark‐based GM analysis of the shell was less reliable, especially when comparing individuals from sympatric sites. Furthermore, neither shell shape nor male genitalia traits allowed for an accurate identification of hybrids between species.

The microsatellite panel was powerful not only to classify individuals correctly into species (>97.5% of the simulated genotypes to each parental group) but also to identify hybrids (from 85% to 97.8% using newhybrids and structure, respectively). Although there was a lower percentage of hybrid genotypes correctly assigned to a specific hybrid class (78%), this was based on 11 loci. The use of additional markers will certainly increase the statistical power to distinguish among different hybrid categories.

The two main mtDNA clades support two divergent lineages that largely correspond to the two species of flat periwinkles. However, shared haplotypes between the two species were observed in substantial proportions, as previously shown by Kemppainen et al. (2009). Although shared haplotypes between species could result from incomplete lineage sorting, introgressive hybridization is supported by three main lines of evidence: (a) hybrids between the two species were detected, including F2s and backcrossed individuals (this study and Carvalho et al., 2016), suggesting not only that hybridization is possible but also that at least some F1s are viable and fertile; (b) a model of divergence with gene flow for this system has a better fit than a model without gene flow (Sotelo et al., 2020); and (c) in contrast with mtDNA, sequences from two nuclear fragments showed no shared haplotypes between the two species (except in Cabo do Mundo) (Sotelo et al., 2020), suggesting that incomplete lineage sorting is unlikely to explain the observed patterns in mtDNA versus nuclear genes.

### 4.2 Extent and patterns of hybridization

The number of locations where hybrids were detected using microsatellites varied between three and seven (depending on the approach), with the majority of hybrids found to be BCO, followed by BCF and F2s. Although no signatures of hybridization were found in most sites, this does not necessarily mean that the two species are completely reproductively isolated at those sites. It is important to emphasize that even if hybrids represent around 5% of individuals in a site, they could have remained undetected with respect to our sample sizes. Nevertheless, this would suggest that hybridization between these species is low in most sites, with the exception of Cabo do Mundo where the highest proportion of hybrids was found (46.9%–56.8%). Several hypotheses may explain the high proportion of hybrids in Cabo do Mundo, and differences among sites in general, such as marginal environmental conditions, low density of snails, and pollution (see Carvalho et al., 2016). Alternatively, the geographic variation in hybridization could reflect a snapshot of temporal variation within sites. However, the exact cause(s) and frequency of hybridization remain elusive.

Our results concerning the *L. fabalis* ecotypes suggest that ecological differences among sites do not have a major influence on hybridization patterns. Although it was not possible to determine with high confidence the ecotype present in the site where a higher number of hybrids was detected, all three ecotypes of *L. fabalis* were found to be involved in hybridization with *L. obtusata* (structure global analysis and mtDNA). This suggests that hybridization occurs even in habitats where *L. obtusata* is less frequent, like those typically occupied by the ME ecotype. Furthermore, the proportion of hybrids involving the same ecotype varies among sites, suggesting that other site‐related characteristics play an important role in determining hybridization. On the other hand, it is possible that hybridization in each site only happens occasionally. Although hybrids were detected in Cabo do Mundo across three different years, long‐term monitoring is needed to evaluate the temporal regularity of hybridization events.

The proportion of sites with signatures of mtDNA introgression is much higher than those with evidence for early generation hybrids using microsatellites. This suggests that hybridization could have been more frequent in the past, prior to the evolution of multiple reproductive barriers, including in currently allopatric sites. However, because mtDNA is unlinked to selected nuclear loci, we cannot exclude that mtDNA introgression can persist and spread over long periods of time without a recent overall diminution of reproductive isolation.

Three lines of evidence suggest that mtDNA introgression is asymmetric in Iberia: (a) a higher proportion of *L. fabalis* populations with signatures of introgression when compared with *L. obtusata* (59% vs. 47%); (b) a slightly higher overall proportion of mtDNA introgression into *L. fabalis* than the other way around (18.5% vs. 16.2%); and (c) individuals from the different hybrid classes show a higher proportion of typical *L. obtusata* over *L. fabalis* haplotypes. However, this asymmetry in Iberia is much weaker than the one observed in Northern Europe, where the overall proportion of mtDNA introgression into *L. fabalis* was 35% versus 6% into *L. obtusata* (Sotelo et al., 2020). Asymmetric mtDNA introgression into *L. fabalis* is in line with previous work showing that males of both species prefer larger females (Saltin, 2013), which suggests that most successful interspecific crosses in Northern European sites preferentially involve a *L. obtusata* female and a *L. fabalis* male. However, different densities of one species relative to the other (or of both species) could also interfere with the choosiness in the field and partially explain differences in the direction of introgression among sites (Carvalho et al., 2016).

### 4.3 Comparison between allopatric and sympatric sites

The number of hybrids and introgressed individuals was clearly higher in sympatric than allopatric locations. This is true whether we consider microsatellites or mtDNA. Concerning recent hybridization (inferred based on microsatellites), there was one *L. fabalis* allopatric site (Mougás) where hybrids were identified (F2), independently of the method. This suggests that either *L. obtusata* was present in that site until recently or *L. obtusata* exists at adjacent sites that were not sampled. Despite being rare, *L. obtusata* can sometimes be found on very exposed shores if protected by rocks from direct wave action (as in Rio de Moinhos, Mindelo, and Viana do Castelo). One additional hybrid was detected in an allopatric *L. obtusata* site (Aldán North), although its classification was not consistent across the different approaches. Since our classification of allopatric and sympatric sites is limited to a specific area and time, our results need to be interpreted with caution. However, because only one site classified as allopatric consistently contained hybrids, our classification of sites was generally reliable.

Individuals with intermediate shell shape and genital morphology were also more common in sympatric sites. However, the degree of shell shape differentiation between individuals of the two species did not vary significantly depending on geographic context (sympatry vs. allopatry). The general lack of correspondence between morphological and genetic hybrids suggests that this intermediate shell shape is not due to current hybridization, although we cannot exclude that later‐generation hybrids, which are harder to detect genetically, could have contributed to the observed pattern. Finally, despite accounting for a smaller component of phenotypic variation, shell morphology in Littorininae is also known to be somewhat plastic (Hollander & Butlin, 2010; Hollander, Collyer, Adams, & Johannesson, 2006; Trussell, 1996, 1997). Thus, phenotypic plasticity could add difficulties in discriminating individuals of each species from the same location using shell shape.

Shell size differences between individuals from the two species are smaller in sympatry than allopatry. We cannot fully exclude an ecotype effect, as most allopatric populations of *L. fabalis* belong to the ME ecotype, which tends to be smaller (i.e., rendering larger size differences between species). However, a similar tendency was observed when only the FI ecotype was considered. Moreover, a trend for smaller size in sympatric populations was observed for *L. obtusata*. Alternatively, this pattern could be explained by increased introgressive hybridization and/or more similar environmental conditions in the sympatric geographic context. However, these results need to be interpreted with caution. Differences between locations, the main factor contributing to shell size variation in this system, could at least partially be explained by individuals’ age. Even though only adult individuals were analyzed, size is known to increase with age. Thus, we cannot exclude that some of the observed patterns in terms of size are heavily influenced by the age at which individuals were collected, which could not be measured.

### 4.4 Hybridization implications for flat periwinkles’ diversification

The relatively high variation among sites in terms of hybridization levels supports the suggestion that the different contacts between flat periwinkle species at local geographical scales can have different evolutionary outcomes. These outcomes depend on factors such as environmental conditions, strength of selection, population densities, and genetic background, among others. Consequently, the degree of reproductive isolation, as well as the reproductive barriers that have accumulated between *L. fabalis* and *L. obtusata*, may well differ among locations but the causes will be hard to disentangle.

Different degrees of reproductive isolation have been observed in other species where multiple replicates of divergence have been studied, as flycatchers (Borge, Lindroos, Nádvorník, Syvänen, & Sætre, 2005), trout (Bettles, Docker, Dufour, & Heath, 2005; Muhlfeld et al., 2014), and lake whitefish (Renaut et al., 2012). Distinct hybridization outcomes among replicates can result from genetic (Borge et al., 2005), behavioral (Bettles et al., 2005), and/or environmental differences (Muhlfeld et al., 2014; Renaut et al., 2012). Environmental change, in particular, has been suggested as the cause of temporal changes in hybridization rates between two trout species, with a recent increase related with changes in precipitation and water temperature (Muhlfeld et al., 2014). At least some of the populations analyzed in this study face marked environmental fluctuations, where exposure to strong wave action may lead to substantial demographic oscillations (Carvalho et al., 2016; Reid, 1996), contributing to temporal instability in potential for hybridization. Furthermore, the recent decline of fucoid macroalgae inhabited by flat periwinkles at the southern limit of their distribution (Nicastro et al., 2013), including the Iberian Peninsula, could contribute to the increase of hybridization in this region.

The fact that hybrids were relatively rare in most of the sites analyzed here suggests that substantial reproductive barriers exist between flat periwinkle species, at least in most sites where they coexist. The difference in penis morphology between flat periwinkles makes this trait an important candidate for playing a role in prezygotic reproductive isolation between these species (Hollander et al., 2013; Reid, 1996). Furthermore, understanding the causes for the high number of hybrids observed in one site and considering whether reinforcement could have evolved in some of these sites are two important issues that need to be addressed in future studies. The possibility of capitalizing on multiple local contacts between *L. fabalis* and *L. obtusata,* with idiosyncratic dynamics, opens exciting research avenues in this system. Performing mating experiments between individuals from sites with varying rates of gene flow between species, phenotypic analyses of traits involved in mating, and the characterization of environmental conditions and species densities among sites are important steps forward to understand the late stages of speciation in this system.

Although the process of divergence between two lineages (forms, ecotypes, or species) tends to be oversimplified as if there was a single possible outcome, the availability of multiple natural replicates suggests that different evolutionary routes are possible. Thus, systems like the one presented here are crucial to understand the different paths that can be taken in the late stages of speciation and to determine which paths are more likely to lead to complete speciation.

## CONFLICT OF INTEREST

None declared.

## AUTHOR CONTRIBUTIONS

DC, GS, and RF designed the study. DC, GS, JH, JC, and RF sampled the individuals. DC, GS, and JC performed the laboratory work; DC, GS, and RF analyzed the genetic data; and DC, AK, JH, and RB analyzed the morphological data. DC, JH, and RF wrote the first draft. All the authors contributed to the final version.

## Data Availability

The new mtDNA haplotype sequences obtained in this study were deposited in GenBank with the accession numbers MN630708–MN630775, whereas the morphological and microsatellite genotypes are publically available in Dryad (https://doi.org/10.5061/dryad.w0vt4b8mk).
