# Supplementary material for: Hybridization patterns between two marine snails, Littorina fabalis and L. obtusata
Source: Ecol Evol. 2020 Jan 21;10(3):1158–79. doi: 10.1002/ece3.5943 (PMC7029087; doi:10.1002/ece3.5943)
Supplement: Supplementary file 1 [file ECE3-10-1158-s001.docx]

**Appendices**

**Appendix 1**

Representation of the typical penis from *Littorina fabalis* and *L. obtusata* (modified from Reid, 1996) showing the features analysed in this study: A, length from tip to end of gland row; B, length of gland row(s); C, penis width where gland row ends; D, number of gland rows; E, number of glands per row; F, total number of glands; G, gland size (x5).


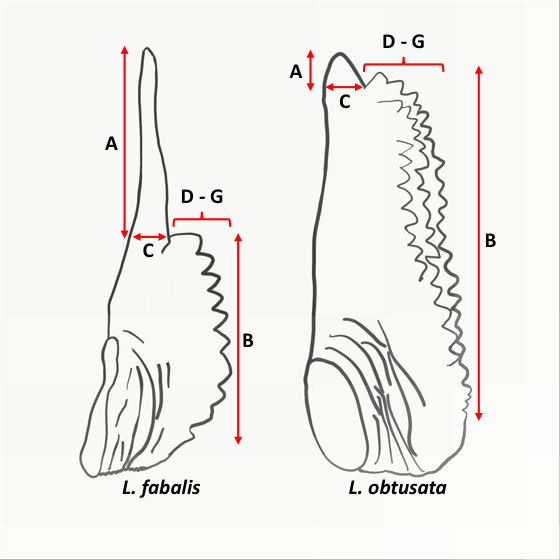


**REFERENCES**

Reid, D.G. (1996). Systematics and evolution of *Littorina*. London, UK: Ray Society.

**Appendix 2**

*Microsatellite loci amplification and quality control*

Amplification was performed in two multiplexes (I and II from Carvalho *et al*. (2016)), with the exception of the *FP3* and *FP7* loci that were replaced by *FP17* and *FP7* in multiplex one and two, respectively. Lack of amplification or ambiguous genotypes were relatively frequent for locus *FP8* in *L. fabalis* samples of the ME ecotype, rendering its exclusion from the dataset. As well, samples (five) displaying more than 20% missing data (i.e. absent information for more than two loci) were removed, resulting in 1059 individuals genotyped for 11 loci were used in subsequent analyses.

In order to assess the quality of the data and exclude putative problematic loci, an initial assignment of individuals to species (or as admixed) based on their multilocus genotype was performed with STRUCTURE v2.3.4 (Pritchard *et al*., 2000; Falush *et al*., 2003, 2007; Hubisz *et al*., 2009). The number of clusters (*k*) was set to 2 (the number of species) and five iterations of 1,000,000 length Monte Carlo Markov Chain (MCMC) runs after a burn-in of 100,000 steps were implemented under a model of admixture and independent allele frequency. All individuals with membership coefficients (*q*) above 0.90 to one of the clusters (putatively pure) were classified into the corresponding species. This dataset was then tested for deviations from Hardy-Weinberg equilibrium (HWE) for each locus/population combination, and linkage disequilibrium (LD) between all pairs of loci for each species across all locations with Genepop v4.2 (Raymond & Rousset, 1995; Rousset, 2008) by means of exact probability tests using a Markov Chain with default parameters. Bonferroni corrections for multiple tests were implemented to adjust for possible false positives. The MICROCHECKER software v2.2.3 (van Oosterhout *et al*., 2004) was posteriorly used to assess the possible causes underlying significant deviations from HWE. Loci that deviated from HWE suggesting genotyping problems, or that were in strong LD suggesting physical linkage, were excluded from subsequent analyses.

Once putative hybrids were removed, three out of 350 HWE tests remained significant after Bonferroni correction (*p*<0.0001): *FP14* for *L. obtusata* from Cabo do Mundo (*F_IS_*=0.7321), *FP2* for *L. fabalis* from Madalena (*F_IS_* =0.4842) and Muros North (*F_IS_* =0.6651), all displaying significant heterozygote deficiency. For *FP2*, the presence of null alleles in those two locations was suggested by the MICROCHECKER analysis. However, since they do not seem to have a widespread effect but are rather more localized; and since it is unlikely that this would affect the general patterns of hybridization in other populations, this locus was maintained for further analyses. Concerning *FP14* in Cabo do Mundo, the MICROCHECKER analysis suggested stuttering, but a careful inspection of the raw data does not support this. Interestingly, significant LD (the only significant test out of 55 after Bonferroni correction, *p*<0.0009) was detected between *FP10* and *FP14* for *L. obtusata* in this same population. A visual inspection of the genotypes of these two loci in this population does not suggest an obvious case of physical linkage, which would also tend to manifest in all locations. Although hybrids (highly frequent in this location) were removed prior testing equilibria, it is possible that signatures of introgressive hybridization were not totally eliminated. Because this may be associated with (real) interactions between alleles from the two species, we decided to maintain the two loci for further analysis.

*Cut-offs to classify individuals as pure or hybrids based on microsatellites*

In order to estimate the STRUCTURE’s membership threshold (*TQ*) to classify individuals as pure or hybrid we simulated 200 genotypes of each parental and hybrid class (F - *L. fabalis*, O - *L. obtusata*, F1 and F2 hybrids, and backcrosses to each parental class - BCF and BCO, respectively) using HYBRIDLAB v1.0 (Nielsen *et al.*, 2006), for both global and local scales. The parental classes of each species (“references”) were simulated based on their real genotypes. For the global-scale analysis, males from allopatric populations with *Q* ≥ 0.99 to one of the species and with concordant penis morphology were selected as references. For the local-scale, references comprised representatives from both species selected from each site. However, due to the generally low number of males, genotypes from females displaying *Q* ≥ 0.99 were also included. Furthermore, in sites where two genetic clusters were identified by STRUCTURE but the number of samples of one of the species was lower than 10, samples from the geographically closest “allopatric” location were added to the input in order to increase power.

All simulated datasets were then used as input to STRUCTURE. For each run, the percentages of pure genotypes misclassified as hybrid and vice versa were calculated for *TQ* values ranging from 0.70 to 1, with increments of 0.02. The graphical representation of these changes allowed us to detect the intersection point where the percentage of individuals incorrectly classified was minimized, thus representing the most appropriate *TQ* (Appendix 3). This was then used to estimate the number of individuals classified as pure or hybrids.

As the procedure to estimate *TQ* cannot be applied for *TPp*, the threshold of the posterior probability obtained with NEWHYBRIDS v1.1 (Anderson & Thompson, 2002), we first calculated the percentage misclassified genotypes as pure from each species or hybrid (independently of the hybrid class assignment) with posterior probabilities ranging from 0.70 to 1, with increments of 0.02 units. Because the number of individuals assigned to a group increases as *Pp* decreases, the *TPp* was chosen as the highest *Pp* value that allowed correct classification of at least 85% of the samples, with the lowest rate of misclassifications.

**REFERENCES**

Anderson, E.C. & Thompson, E.A. (2002). A model–based method for identifying species

hybrids using multilocus genetic data. Genetics, 160(3), 1217–1229.

Falush, D., Stephens, M. & Pritchard, J.K. (2007). Inference of population structure using

multilocus genotype data: dominant markers and null alleles. Molecular Ecology Notes, 7(4), 574-578. https://doi.org/10.1111/j.1471-8286.2007.01758.x

Falush, D., Stephens, M. & Pritchard, J.K. (2003). Inference of population structure using

multilocus genotype data: linked loci and correlated allele frequencies. Genetics, 164(4), 1567-1587.

Hubisz, M.J., Falush, D., Stephens, M. & Pritchard, J.K. (2009). Inferring weak population structure with the assistance of sample group information. Molecular Ecology Resources,

9(5), 1322-1332. https://doi.org/10.1111/j.1755-0998.2009.02591.x

Nielsen, E.E., Bach, L.A. & Kotlicki, P. (2006). HYBRIDLAB (version 1.0): a program for

generating simulated hybrids from population samples. Molecular Ecology Notes, 6(4),

971-973. https://doi.org/10.1111/j.1471-8286.2006.01433.x

Pritchard, J.K., Stephens, M. & Donnelly, P. (2000). Inference of population structure using

multilocus genotype data. Genetics, 155(2), 945-959.

Raymond, M., & Rousset, F. (1995). GENEPOP (Version 1.2): Population genetics software for exact tests and ecumenicism. Journal of Heredity, 86, 248-249. https://doi.org/10.1093/oxfordjournals.jhered.a111573

Rousset, F. (2008). GENEPOP’007: a complete re‐implementation of the GENEPOP software for Windows and Linux. Molecular Ecology Resources, 8(1), 103-106. https://doi.org/10.1111/j.1471-8286.2007.01931.x

van Oosterhout, C., Hutchinson, W.F., Wills, D.P.M. & Shipley, P. (2004). Micro-Checker: Software for Identifying and Correcting Genotyping Errors in Microsatellite Data. Molecular Ecology Notes, 4(3), 535-538. https://doi.org/10.1111/j.1471-8286.2004.00684.x

**Appendix 3**.

Graphical representation of the method to estimate the threshold *Q* value (T*Q*) for individual assignment to species (*i.e*. pure) or hybrid according to STRUCTURE analysis (for *k*=2) of the simulated dataset. For a given *Q* value, from 0.70 to 1 (on the x axis), the percentage of misclassified genotypes (on the y axis) for pure (blue line) and hybrid (red line) classes is represented. T*Q* is defined by the intersection of the two lines (0.90), i.e. where the percentage of misclassification is minimized.

**Appendix 4**.

Minimum membership threshold used to classify individuals as pure or hybrid based on STRUCTURE for the local approach. Due to low sample sizes of one species in some sites, samples were grouped (+) with those from a close by site (see Material and Methods).

| **Location** | **T*Q*** |
| --- | --- |
| Burela + Morás | 0.92 |
| Abelleira + Muros North | 0.90 |
| Lanzada North | 0.94 |
| Seixiños | 0.92 |
| Aldán North + Aldán South | 0.92 |
| Borna | 0.92 |
| Cangas | 0.92 |
| La Guia | 0.90 |
| Alcabre | 0.92 |
| Redondela + Mougás | 0.88 |
| Mindelo | 0.92 |
| Cabo do Mundo | 0.94 |
